# Are We on the Right Track: Can Our Understanding of Abscission in Model Systems Promote or Derail Making Improvements in Less Studied Crops?

**DOI:** 10.3389/fpls.2015.01268

**Published:** 2016-01-26

**Authors:** Sara E. Patterson, Jenny L. Bolivar-Medina, Tanya G. Falbel, Janet L. Hedtcke, Danielle Nevarez-McBride, Andrew F. Maule, Juan E. Zalapa

**Affiliations:** ^1^Department of Horticulture, University of Wisconsin–MadisonMadison, WI, USA; ^2^Vegetable Crops Research Unit, United States Department of Agriculture – Agricultural Research ServiceMadison, WI, USA; ^3^West Madison Agricultural Research StationVerona, WI, USA

**Keywords:** abscission, shedding, seed-shatter, grape, cranberry, fonio, tomato

## Abstract

As the world population grows and resources and climate conditions change, crop improvement continues to be one of the most important challenges for agriculturalists. The yield and quality of many crops is affected by abscission or shattering, and environmental stresses often hasten or alter the abscission process. Understanding this process can not only lead to genetic improvement, but also changes in cultural practices and management that will contribute to higher yields, improved quality and greater sustainability. As plant scientists, we have learned significant amounts about this process through the study of model plants such as *Arabidopsis*, tomato, rice, and maize. While these model systems have provided significant valuable information, we are sometimes challenged to use this knowledge effectively as variables including the economic value of the crop, the uniformity of the crop, ploidy levels, flowering and crossing mechanisms, ethylene responses, cultural requirements, responses to changes in environment, and cellular and tissue specific morphological differences can significantly influence outcomes. The value of genomic resources for lesser-studied crops such as cranberries and grapes and the orphan crop fonio will also be considered.

## Introduction

**FIGURE 1:**
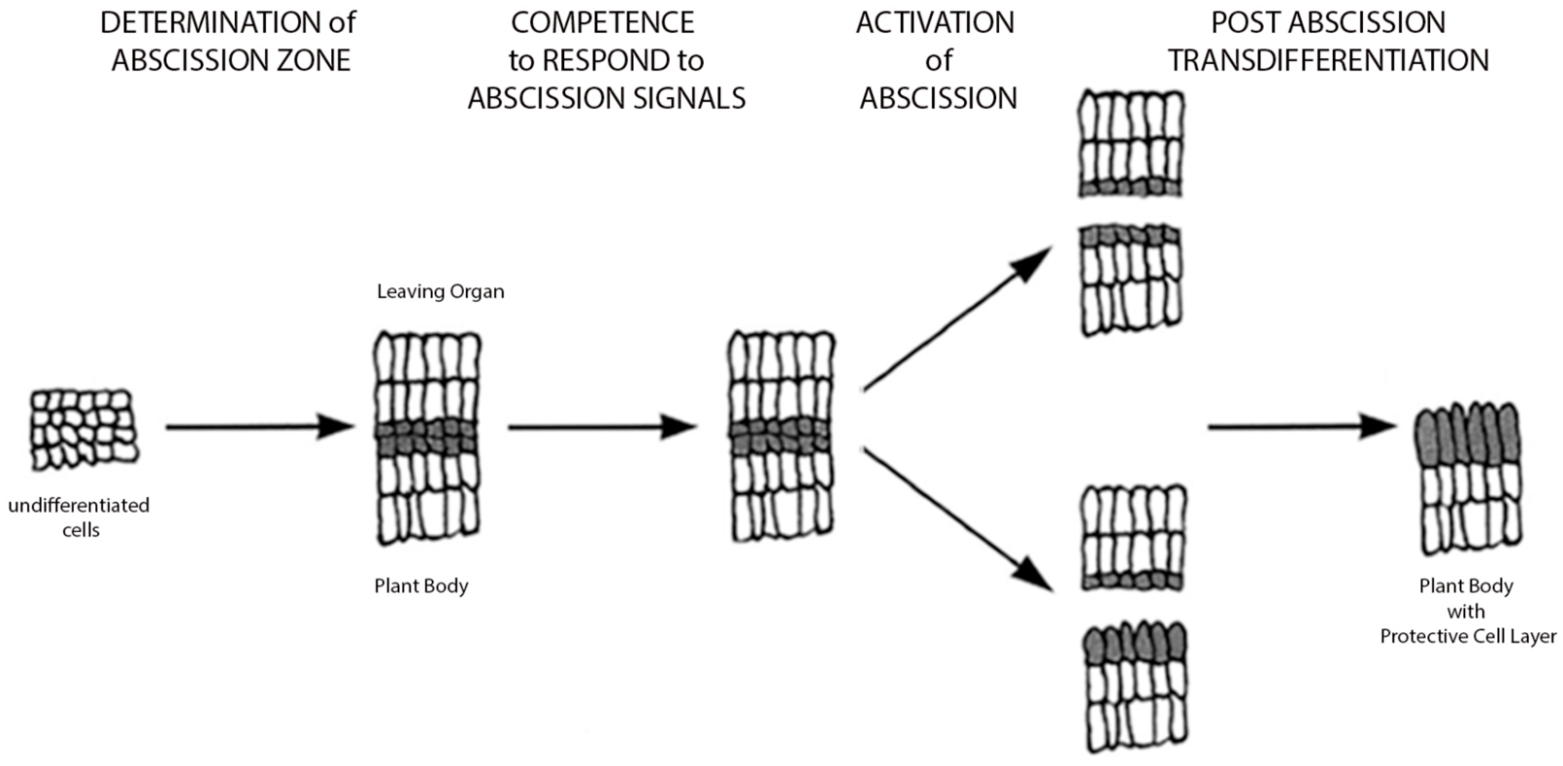
**Model of abscission**. Simple schematic depicting process of abscission (adapted from Patterson, 2001). Four basic stages are depicted: Stage 1: Determination of the abscission zone, Stage 2: Competence of abscission zone to respond to abscission signals, Stage 3: Activation of abscission or cell separation event leading to organ detachment, and Stage 4: Transdifferentiation of the proximal layer creating a protective layer for plant. (For a more comprehensive model with gene pathways, see Estornell et al., 2013).

Historically, humans have selected crop plants with delayed abscission for generations, as early fruit drop or seed shatter limited effective collection of the fruits, grains, or legumes (Harlan, 1992; Plants and Society, 2006). In general, we know the process of abscission results in shedding of organs as a developmentally programmed event; however, abscission may also occur in response to pathogens, environmental cues or other stresses. Early studies on abscission focused on the anatomical and physiological characterization of the abscission zone (Addicott, 1982; Sexton and Roberts, 1982). These studies have shown that the abscission zone consists of a few to multiple cell layers and is distinguished by small densely cytoplasmic cells. During the abscission process, there is breakdown of the middle lamella of cells within the separation layers. Although historically, there have been several proposed models for genes regulating abscission, scientists are still unclear as what are the key players and how plant hormones, like ethylene, jasmonic acid (JA), abscisic acid, and auxin affect the regulation of gene expression during the process (**Figure 1**). There is strong evidence for interplay between the plant hormones ethylene and auxin in regulating abscission timing, where the former enhances the process and the latter inhibits (Abeles and Rubinstein, 1964; Jensen and Valdovinos, 1967; Addicott, 1982; Osborne, 1989). In addition, early researchers also focused on elucidating the role of cell degrading enzymes including the polygalacturonases and cellulases during the abscission process (Abeles, 1969; del Campillo and Bennett, 1996; del Campillo, 1999). Model systems including rice, maize, *Arabidopsis*, and tomato have provided new valuable genetic information on abscission and shattering, and knowledge of these genes associated with abscission has the potential to radically change approaches to studying abscission (Lewis et al., 2006; Estornell et al., 2013; Niederhuth et al., 2013).

### Abscission in Monocots

In the grasses (maize, sorghum, and rice), initially two transcription factors were identified as associated with the regulation of shattering, *qSH1* and *SH4* (Konishi et al., 2006; Li et al., 2006). *SHAT1*, an *APETELA2* transcription factor, has also been identified as a gene that also affects shattering (Zhou et al., 2012). These genes are key factors in eliminating most shattering in members of the Poaceae family. In addition, homeodomain-leucine zipper transcription factors (HD-Zip TFs) and genes associated with growth regulation including auxin and ethylene responses from sorghum and maize are expressed in floral abscission zones (Chew et al., 2013; Dwivedi et al., 2014). Most recently, BRITTLE RACHIS1 and 2 were identified in barley and shown to be responsible for seed shatter (Pourkheirandish et al., 2015). Especially interesting is the fact that BRI1, 2 are hypothesized to act as receptor and ligand and BRI2 has been shown to have homology with the *Arabidopsis* protein IDA that is also hypothesized as a receptor ligand (Butenko et al., 2003; Pourkheirandish et al., 2015). Orphan grain crops such as *Digitaria exilis* (fonio), *Eragrostis tef* (teff), and *Eleusine coracana* (finger millet) often have major losses due to early or unregulated shattering, and thus could highly benefit from breeding for delayed abscission and abscission associated genes through introgression of favorable alleles. While genes such as *qSH1*, *SH4*, and *SHAT1* have been shown to regulate shattering in domesticated rice, recent studies also show that panicle structure may also be critical (Ishii et al., 2013). Thus, undue attention to only specific genes or a single trait rather than multiple gene traits, might result in less effective selection.

### Abscission in Dicots: *Arabidopsis* as Model System

In dicots, *Arabidopsis* has served as the model system to study abscission, and researchers have gained significant insights concerning regulation of the abscission process. Genes regulating development of the abscission zone and responses to hormonal, environmental, and newly discovered endogenous signals regulating abscission have been extensively studied. There are many excellent reviews: (Roberts et al., 2000, 2002; Aalen et al., 2006, 2013; Binder and Patterson, 2009; Van Nocker, 2009; Liljegren, 2012; Estornell et al., 2013; Niederhuth et al., 2013). While considerable inroads have been made on understanding the genes involved in signaling, the exact pathways are still being defined (Liljegren, 2012; Niederhuth et al., 2013). These key players include *IDA* (Butenko et al., 2003, 2009; Stenvik et al., 2008), *HAESA* (Jinn et al., 2000), *HAESA LIKE* (Shi et al., 2011), *NEVERSHED* (Liljegren et al., 2009), and *EVERSHED* (Leslie et al., 2010). Additional downstream signaling factors have also been identified and include *SERK1*, *BREVIPEDICELLUS/KNOTTED-LIKE FROM ARABIDOPSIS THALIANA* (*BP/KNAT1*; Wang et al., 2006; Shi et al., 2011), and MAP kinases (Meng et al., 2012). Genes that are critical for formation of the abscission zone in *Arabidopsis* include *BOP1* and *BOP2*, and the *MADS BOX* gene *AGL15* (Fernandez et al., 2000; Hepworth et al., 2005; McKim et al., 2008).

Additional transcription factors that have been identified include *FOREVER YOUNG FLOWER* (*FYF*; Chen et al., 2011) and the zinc finger protein *Arabidopsis ZINC ZINGER PROTEIN 2* (Cai and Lashbrook, 2008). While these genes have been shown to be involved in the abscission process, the actual function during the abscission process is quite undefined. Similarly, genes regulating organ boundary patterning and elasticity of boundaries have been identified and while many have no determined role, the F-box gene *HAWAIIAN SKIRT* has been shown to disrupt normal patterning leading to fusion of sepals, and consequently delayed abscission (Aida and Tasaka, 2006; Gonzalez-Carranza et al., 2007b; Rast and Simon, 2008). An additional F-box gene *COI1* also delays abscission; however, it has been determined that this delay is most likely due to altered regulation of ethylene and auxin responses during the process of abscission in response to the absence of JA signaling rather than formation of the abscission zone (Kim et al., 2013). And, while the role of JA during abscission was initially a surprise, the role of other hormones such as ethylene and auxin during abscission has been well characterized in *Arabidopsis* (Patterson and Bleecker, 2004; Ellis et al., 2005; Binder and Patterson, 2009; Ogawa et al., 2009; Basu et al., 2013; Kim, 2014). These include ethylene synthesis genes (*ALLENE OXIDE SYNTHESIS*), ethylene response genes (*ETR1, EIN2*, and *EIN3*) and auxin-associated genes (*ARF1, ARF2*, and *AUX1*).

Many genes regulating cell wall modifications have also been identified and studied for their role in abscission in *Arabidopsis* as well as other species. These include polygalacturonases (Gonzalez-Carranza et al., 2002, 2007a; Kim and Patterson, 2006; Kim et al., 2006), cellulases (del Campillo, 1999), expansions (Cho and Cosgrove, 2000; Lashbrook and Cai, 2008), pectate lyases, xyloglucans and glycosylase transferases (Lashbrook and Cai, 2008; Wei et al., 2010; Singh et al., 2011). In addition, determination of unique morphological characteristics of the abscission zone have been characterized by multiple research groups: cell number, scar formation, timing and the relationship to environmental stresses, and developmental processes such as pollination, fertility, and senescence (Sawicki et al., 2015). There are also new studies indicating that alkalization of the cytosol of cells within the abscission zone is particularly important (Sundaresan et al., 2015). Last, cell death markers including *LZ ribonuclease* and *BFN1 nuclease* have been characterized for their roles during the abscission process (Farage-Barhom et al., 2008; Bar-Dror et al., 2011). In summary, there are many genes identified in *Arabidopsis* that impact the process of abscission and additional research will be needed before all the key players are characterized.

### Abscission in Dicots: Tomato as Model System

**FIGURE 2:**
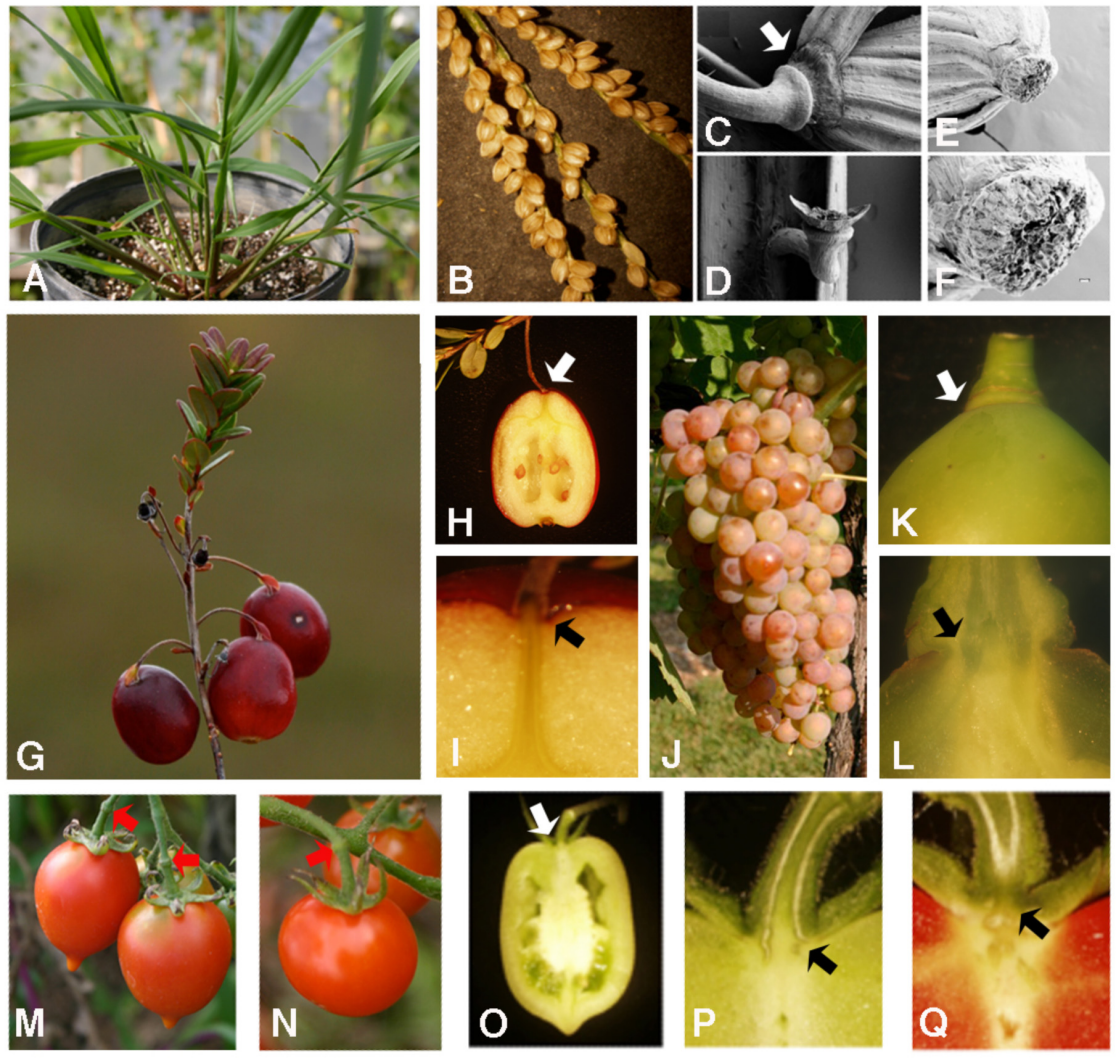
**Abscission in fonio, cranberry, grapes, and tomatoes. (A–F)** Images of fonio illustrating the developing plant **(A)**, a mature inflorescence **(B)**, and scanning electron micrographs of the abscission zone of a mature seed **(C–F)**. **(G–I)** Images of cranberry illustrating the developing fruit **(G)** and sections revealing the fruit abscission zone **(H,I)**. **(J–L)** Images of grape illustrating a fruit cluster **(J)** and sections revealing the fruit abscission zone **(K,L)**. **(M–Q)** Images of tomatoes illustrating the abscission zones in tomatoes. A variety of cultivars were observed and pictured are two cluster varieties: Principe Borghese **(M)**, and Ladybug **(N)**. **(O–Q)** Illustrate freehand sections of the pedicel abscission zone attached directly to the developing fruit. Black and white arrows show fruit/pedicel abscission zones and red arrows show the “knuckle” abscission zone.

Abscission in tomato has also been studied quite extensively, as tomato has been considered a model crop that is relatively easy to work with: true breeding (self pollinated), moderate sized genome (900 Mb), excellent isogenic stock collections, well characterized genetics, excellent physiological research studies, and easily transformed. Genome information, gene expression, and information about isogenic genetic stocks are available through the tomato functional genomics database (TFGD), the Sol Genomics network (http://solgenomics.net/) and NCBI. While there is considerable knowledge about genes regulating abscission within the pedicel in jointed tomatoes *JOINTLESS* (Mao et al., 2000; Nakano et al., 2013; Guan et al., 2014; Ito and Nakano, 2015), there is still relatively little understood concerning regulation of abscission at the fruit pedicle junction. These distinctions are valuable in terms of marketing different types of tomatoes (cluster on the vine versus slicing) and in shipping. In jointed tomatoes (see **Figure 2**) abscission within the pedicel or at the knuckle has been shown to be regulated by numerous MADS Box genes, auxin associated genes and several novel transcription factors (Mao et al., 2000; Nakano et al., 2013; Guan et al., 2014; Ito and Nakano, 2015; Ma et al., 2015). Researchers have also extensively studied the role of cell wall hydrolytic enzymes and polysaccharides including cellulases, polygalacturonases, pectinases, xyloglucans, arabinans, and galactans during tomato fruit abscission (del Campillo and Bennett, 1996; Kalaitzis et al., 1997; Iwai et al., 2013). Many of the cell-wall associated genes are members of large gene families; and thus, efforts to alter the abscission process through modification of these genes have not yielded significant changes. In addition not all ethylene associated genes that have been shown to affect abscission in *Arabidopsis* delay abscission in tomato. While mutations in *ETR1, ETR2*, and *ETR3* all delay the process of abscission in *Arabidopsis*, only the mutated ortholog of *ETR1 (NEVERRIPE*) delays abscission in tomato; and they all are primarily associated with fruit ripening (Lanahan et al., 1994; Klee, 2002). Historically, breeders have focused on fruit size, color, and flavor rather than abscission. Perhaps the marketing of increased tomato varieties in markets and the new emphasis on local produce, abscission in cluster tomatoes may warrant further study on abscission in tomatoes.

### Approaches to Study Abscission in Both the Grasses and Dicots

Many of the genes found in maize, rice, and *Arabidopsis* are highly conserved and plant breeders have embraced this knowledge to direct research programs toward utilizing genomic approaches. With the tools and knowledge to identify and engineer alternative plant species, will these efforts prove productive? There is no doubt that as the world population grows and resources and climate conditions change, it is critical that we continue to increase crop production and develop more sustainable agricultural practices. While there are 100s of crops to consider, this manuscript will use examples such as tomato (an annual vegetable crop), grapes (a perennial fruit tree crop), cranberries (a perennial fruit crop), and fonio (an annual grass native to West Africa). These crops have been selected to provide a perspective on both well-studied crops and less developed crops (**Figure 2**).

### Crop Examples

#### Fonio – An Orphan Grass: Challenges and Success

Our major grain crops including maize, wheat, and barley have been selected for delayed abscission by early gatherers for thousands of years. In addition, some millet varieties that have been cultivated for centuries have reduced shatter (Zohary and Hopf, 2000; Brink and Belay, 2006). However, uncontrolled seed shatter in fonio *Digitaria exilis* and many of the orphan grain crops is one of the growers’ greatest concerns and a major limiting factor in their potential for expansion as food crops. These orphan grain crops are often drought and disease resistant and small plots are routinely grown in many communities. Addressing seed shattering in crops such as fonio could increase yields by more than a third, providing farmers significant additional income (Foltz, personal communication). Of major importance is that this could be done with out adding fertilizer. In contrast to other commonly grown grains, fonio requires little or no inputs; thus not requiring additional investment by farmers. For example, in Mali, yields of common millet could be improved with fertilizer applications, yet this would require investment; and, predicted profits from a 50% increase in yield due to these added resources would not exceed the predicted profits from improved fonio with reduced shattering. As the world population grows and climate conditions change, resource management is critical and thus attention to breeding and management practices for small orphan grains has become extremely important.

Currently, fonio is grown extensively in Guinea, Gambia, Senegal, Mali, Burkina Faso, Benin, Senegal, Togo, Nigeria, and the Dominican Republic. In most of West Africa, fonio is grown primarily by smallholders on plots typically of one hectare or less; and harvest must be performed within a couple days of full grain development due to seed shatter. Abscission, or seed shatter in fonio has not been studied in the past; and thus, one of the first challenges has been to characterize flowering and the abscission process. We have observed that flowering in fonio is tightly regulated, as initially reported by Kwon-Ndung and Misari (1999), Cruz (2004), Cruz et al. (2006), Tekete (2006), and Cruz and Béavogui (2011). This photoperiodic control of flowering and seed development results in the narrow window for harvest. We also believe that abscission in fonio is both environmentally and developmentally regulated, and grains abscise with the first heavy rainfall after reaching full maturity. These observations propelled us to identify genetic factors regulating abscission as modification of weather or flowering time was less achievable.

Since there was initially no sequence data on fonio, we performed a single Illumina sequence run to generate a snapshot of the transcriptome of a Niatia seedling (one of the most common commercially available lines). The run yielded 38 million reads that we mapped directly onto to the rice genome and the rice transcriptome, as well as assembled *de novo.* We aligned contigs from the transcript assembly with the sequence of several known shattering genes and their homologs in maize, rice, and sorghum, and found extensive fonio sequence similarities with the rice shattering gene *qSH1*, members of the Agamous-related family of genes such as *JOINTLESS 1-3*, *SH2*, and *SH3*, the free-threshing locus *Q* of wheat, as well as the *Arabidopsis* abscission-associated gene *NEVERSHED* (Liljegren et al., 2009; Sang, 2009). Using this sequence information we have been able to PCR amplify portions of the *qSH1* homolog from fonio genomic DNA. While this provides proof of principle, it still needs to be determined if selection for mutants in *qSH1* would provide a delay in seed shatter. As fonio seed is quite small like *Arabidopsis*, a targeted approach to screen mutagenized seed similar to iTILLING could be utilized (Bush and Krysan, 2010).

Overall, there are gains to be made through targeted gene selection in fonio and other small orphan grains; but the challenges still remain. Selection of cultivars/lines to study is one of the first questions, as historically each community has their own local lines. Some lines are diploid and others thought to be tetraploid. In addition, fonio is self-pollinated and potentially apomictic; and thus, crosses between lines are challenging. Techniques for pollination of finger millet have been developed by the Devos and Bennetzen labs at the University of Georgia and are being applied to crossing fonio. Communication with remote villages and farmers as well as distribution of new seed and management practices will also be challenging. However, despite these concerns, crops like fonio have significant potential for increases in yield by understanding the abscission process and breeding for genes regulating seed shatter.

#### Woody Perennial Dicots: Cranberries and Grapes-Challenges and Successes

Historically berry drop or abscission has not been a trait that either grape or cranberry growers have focused on improving. However, with the recent focus on conservation of water and resources and the introduction of new cold-hardy hybrid grapes (*Vitis vinifera* × *Vitis riparia* and *Vitis vinifera* ×* Vitis labrusca*) berry drop has become an issue in both crops. With wine grapes, early abscission results in lower sugar content and higher acidity, and thus a poorer quality wine in general. Alternatively, in table grapes it is even more important to retain high quality full clusters after harvest. Uncontrolled berry drop decreases both value and quality. Curiously, increased berry drop in some cultivars such as “Sunpreme” may prove fruitful for raisin producers, as some growers are now taking advantage of berry abscission to reduce harvest costs (Romero, 2015). In cranberry, there has been minimal research regarding abscission, but it is believed that cultural and environmental factors such as limited nutrient availability and extreme heat conditions can cause fruit drop. Growers have placed a new emphasis on management of water and sustainable production in response to climate change and new environmental stresses. Consequently, the loss of fruit is an important issue as fruit growth and abscission is most likely dependent on transport of water, nutrients and other factors across this zone (Sawicki et al., 2015).

We searched available databases for both grapes and cranberries for orthologs to 15 genes previously identified in *Arabidopsis* that have been characterized as regulating the abscission process or associated with unique stages of development in the abscission process (Supplementary Table S1). In grapes (*Vitis vinifera*), we selected orthologs for ten genes (NCBI); and are currently looking at gene expression during the abscission process in four hybrid cultivars of cold-hardy Wisconsin grapes. In cranberry, joint efforts across the United States have recently yielded a transcriptome and nuclear genome assembly (Polashock et al., 2014) and a nuclear genome assembly (Fajardo et al., 2014). We used both of these cranberry generated databases to search for abscission genes. While matches for all fifteen of the genes we searched for were identified in both grape and cranberry genomes, too many close matches to identify a single ortholog was frequently an issue (Supplementary Table S1). In general, the high homology of both cranberry and grape genes to known abscission-related genes from *Arabidopsis* suggests that there may be shared functions and similar signaling pathways regulating the abscission process. Identification of the best candidate ortholog, transcription factors and unique aspects of development associated with each species may make altering abscission more challenging. Ultimately, improving our understanding of both early and late fruit abscission in these fruit crops using molecular tools combined with traditional breeding, morphological and physiological studies will lead to better management practices and improved quality and greater yields.

## Summary: Why the Success in Some Crops and Not Others?

Building our knowledge on abscission in crop plants continues to be an important challenge not only not to prevent unwanted abscission but also to promote early abscission, as in many cases early bud removal and accelerated fruit abscission promotes improved root development, a more vigorous plant, and higher quality fruit and flowers. The rapid advances in molecular techniques and availability of quality sequence information on most species has spurred interest and promoted new research on the cloning and engineering known genes. While many of these genes will definitely have similar functions in many crops, researchers must always pause and remember the developmental biology of their plant such as flowering time, pollination, fertility, fruit development, life cycle, and senescence. Crops may vary as to whether they abscise at the abscission zone associated directly with the fruit or at an independent zone within the pedicel; and thus knowing the biology and marketing traits of the crop must be considered. In addition, developmental programs may mask other traits; and thus, a gene altering the abscission process may have no effect in specific genetic backgrounds. This is the case in the recently discovered role of panicle structure in rice and the hidden role in abscission (Ishii et al., 2013). Clearly, it is essential that breeders and molecular biologists work together providing an understanding of the unique development of each species as well as the targeted genes or pathways of interest. Similarly, it will also be critical to consider genome size, ploidy, and genetic relationships amongst lines as well as between species. Our progress may be slow at times; but a concerted combined effort promises new insights.

## Author Contributions

AM, JB-M, JH, DN-M, TF, JZ, and SP designed the experiments. AM, JB-M, DN-M, TF, and SP performed the experiments. SP and JZ wrote and edited the manuscript. In addition, all authors contributed to editing the manuscript.

## Conflict of Interest Statement

The authors declare that the research was conducted in the absence of any commercial or financial relationships that could be construed as a potential conflict of interest.

## References

[B1] Aalen R. B. Butenko M. A. Stenvik G.-E. Tandstad N. M. Patterson S. E. (2006). “Genetic control of floral abscission,” in *Floriculture, Ornamental and Plant Biotechnology: Advances and Topical Issues*, ed. Silva J.T.d (London: Global Science Books Ltd.), 101–108.

[B2] Aalen R. B. Wildhagen M. Stø I. M. Butenko M. A. (2013). IDA: a peptide ligand regulating cell separation processes in *Arabidopsis*. *J. Exp. Bot.* 64 5253–5261. 10.1093/jxb/ert338 24151306

[B3] Abeles F. B. (1969). Abscission: role of cellulase. *Plant Physiol.* 44 447–452. 10.1104/pp.44.3.447 16657082 PMC396106

[B4] Abeles F. B. Rubinstein B. (1964). Regulation of ethylene evolution and leaf abscission by auxin. *Plant Physiol.* 39 963–969. 10.1104/pp.39.6.963 16656043 PMC550201

[B5] Addicott F. (1982). *Abscission.* Berkeley, CA: University of California Press.

[B6] Aida M. Tasaka M. (2006). Genetic control of shoot organ boundaries. *Curr. Opin. Plant Biol.* 9 72–77. 10.1016/j.pbi.2005.11.011 16337829

[B7] Bar-Dror T. Dermastia M. Kladnik A. Znidaric M. T. Novak M. P. Meir S. (2011). Programmed cell death occurs asymmetrically during abscission in tomato. *Plant Cell* 23 4146–4163. 10.1105/tpc.111.092494 22128123 PMC3246325

[B8] Basu M. M. González-Carranza Z. H. Azam-Ali S. Tang S. Shahid A. A. Roberts J. A. (2013). The manipulation of auxin in the abscission zone cells of *Arabidopsis* flowers reveals that indoleacetic acid signaling is a prerequisite for organ shedding. *Plant Physiol.* 162 96–106. 10.1104/pp.113.216234 23509178 PMC3641234

[B9] Binder B. Patterson S. (2009). Ethylene-dependent and –independent regulation of abscission. *Stewart Postharvest Rev.* 5 1–10. 10.2212/spr.2009.1.1

[B10] Brink M. Belay G. (2006). *Plant Resources of Tropical Africa I: Cereals and Pulses.* Wageningen: PROTA Foundation, 54–57.

[B11] Bush S. M. Krysan P. J. (2010). iTILLING: a personalized approach to the identification of induced mutations in *Arabidopsis*. *Plant Physiol.* 154 25–35. 10.1104/pp.110.159897 20668060 PMC2938168

[B12] Butenko M. A. Patterson S. E. Grini P. E. Stenvik G. E. Amundsen S. S. Mandal A. (2003). Inflorescence deficient in abscission controls floral organ abscission in *Arabidopsis* and identifies a novel family of putative ligands in plants. *Plant Cell* 15 2296–2307. 10.1105/tpc.014365 12972671 PMC197296

[B13] Butenko M. A. Vie A. K. Brembu T. Aalen R. B. Bones A. M. (2009). Plant peptides in signalling: looking for new partners. *Trends Plant Sci.* 14 255–263. 10.1016/j.tplants.2009.02.002 19362511

[B14] Cai S. Lashbrook C. C. (2008). Stamen abscission zone transcriptome profiling reveals new candidates for abscission control: enhanced retention of oral organs in transgenic plants overexpressing *Arabidopsis* ZINC FINGER PROTEIN2. *Plant Physiol.* 146 1305–1321. 10.1104/pp.107.110908 18192438 PMC2259061

[B15] Chen M. K. Hsu W. H. Lee P. F. Thiruvengadam M. Chen H. L. Yang C. H. (2011). The MADS box gene, FOREVER YOUNG FLOWER, acts as a repressor controlling floral organ senescence and abscission in *Arabidopsis*. *Plant J.* 68 168–185. 10.1111/j.1365-313X.2011.04677.x 21689171

[B16] Chew W. Hrmova M. Lopato S. (2013). Role of homeodomain leucine zipper (HD-Zip) IV transcription factors in plant development and plant protection from deleterious environmental factors. *Int. J. Mol. Sci.* 14 8122–8147. 10.3390/ijms14048122 23584027 PMC3645734

[B17] Cho H. T. Cosgrove D. J. (2000). Altered expression of expansin modulates leaf growth and pedicel. *Proc. Natl. Acad. Sci. U.S.A.* 97 9783–9788. 10.1073/pnas.160276997 10931949 PMC16942

[B18] Cruz J. F. (2004). Fonio: a small grain with potential. *LEISA Magazine* 20 16–17.

[B19] Cruz J. F. Béavogui F. (2011). *Le Fonio, une Céréale Africaine.* Versailles: Quae.

[B20] Cruz J. F. Drame D. Kouyate S. Marouzé C. Sakho S. Son G. (2006). “Improvement of fonio post-harvest technology. Mechanization of processing operations,” in *Proceedings of Technological Innovation and Enhancement of Marginal Products: Foggia, 6-8 April 2005*, eds Severini C. De Pilli T. Giuliani R. (Foggia: Claudio Grenzi), 256–257.

[B21] del Campillo E. (1999). Multiple endo-1,4-beta-D-glucanase (cellulase) genes in *Arabidopsis*. *Curr. Top. Dev. Biol.* 46 39–61. 10.1016/S0070-2153(08)60325-7 10417876

[B22] del Campillo E. Bennett A. B. (1996). Pedicel break strength and cellulase gene expression during tomato flower abscission. *Plant Physiol.* 111 813–820. 10.1104/pp.111.3.813 8754682 PMC157899

[B23] Dwivedi K. K. Roche D. J. Clemente T. E. Ge Z. Carman J. G. (2014). The OCL3 promoter from *Sorghum bicolor* directs gene expression to abscission and nutrient-transfer zones at the bases of floral organs. *Ann. Bot.* 114 489–498. 10.1093/aob/mcu148 25081518 PMC4204675

[B24] Ellis C. M. Nagpal P. Young J. C. Hagen G. Guilfoyle T. J. Reed J. W. (2005). AUXIN RESPONSE FACTOR1 and AUXIN RESPONSE FACTOR2 regulate senescence and floral organ abscission in *Arabidopsis thaliana*. *Development* 132 4563–4574. 10.1242/dev.02012 16176952

[B25] Estornell L. H. Agusti J. Merelo P. Talon M. Tadeo F. R. (2013). Elucidating mechanisms underlying organ abscission. *Plant Sci.* 19 48–60. 10.1016/j.plantsci.2012.10.008 23265318

[B26] Fajardo D. Schlautman B. Steffan S. Polashock J. Vorsa N. Zalapa J. (2014). The American cranberry mitochondrial genome reveals the presence of selenocysteine (tRNA-Sec and SECIS) insertion machinery in land plants. *Gene* 536 336–343. 10.1016/j.gene.2013.11.104 24342657

[B27] Farage-Barhom S. Burd S. Sonego L. Perl-Treves R. Lers A. (2008). Expression analysis of the BFN1 nuclease gene promoter during senescence, abscission, and programmed cell death-related processes. *J. Exp. Bot.* 59 3247–3258. 10.1093/jxb/ern176 18603613 PMC2529240

[B28] Fernandez D. E. Heck G. R. Perry S. E. Patterson S. E. Bleecker A. B. Fang S. C. (2000). The embryo MADS domain factor AGL15 acts postembryonically. Inhibition of perianth senescence and abscission via constitutive expression. *Plant Cell* 12 183–198. 10.1105/tpc.12.2.183 10662856 PMC139757

[B29] Gonzalez-Carranza Z. H. Elliott K. A. Roberts J. A. (2007a). Expression of polygalacturonases and evidence to support their role during cell separation processes in *Arabidopsis thaliana*. *J. Exp. Bot.* 58 3719–3730. 10.1093/jxb/erm222 17928369

[B30] Gonzalez-Carranza Z. H. Rompa U. Peters J. L. Bhatt A. M. Wagstaff C. Stead A. D. (2007b). HAWAIIAN SKIRT: an F-box gene that regulates organ fusion and growth in *Arabidopsis*. *Plant Physiol.* 144 1370–1382. 10.1104/pp.106.092288 17496113 PMC1914148

[B31] Gonzalez-Carranza Z. Whitelaw C. Swarup R. Roberts J. (2002). Temporal and spatial expression of a polygalacturonase during leaf and flower abscission in oilseed rape and *Arabidopsis*. *Plant Physiol.* 128 534–543. 10.1104/pp.010610 11842157 PMC148916

[B32] Guan X. Xu T. Gao S. Qi M. Wang Y. Xin L. (2014). Temporal and spatial distribution of auxin response factor genes during tomato flower abscission. *J. Plant Growth Regul.* 33 317–327. 10.1007/s00344-013-9377-x

[B33] Harlan J. R. (1992). *Crops and Man*, 2nd Edn Madison, WI: American Society of Agronomy and Crop Science Society of America.

[B34] Hepworth S. R. Zhang Y. McKim S. Li X. Haughn G. W. (2005). BLADE-ON-PETIOLE-dependent signaling controls leaf and floral patterning in *Arabidopsis*. *Plant Cell* 17 1434–1448. 10.1105/tpc.104.030536 15805484 PMC1091766

[B35] Ishii T. Numaguchi K. Miura K. Yoshida K. Thanh P. T. Htun T. M. (2013). OsLG1 regulates a closed panicle trait in domesticated rice. *Nat. Genet.* 45 462–465. 10.1038/ng.2567 23435087

[B36] Ito Y. Nakano T. (2015). Development and regulation of pedicel abscission in tomato. *Front. Plant Sci.* 6:442 10.3389/fpls.2015.00442 PMC4462994 26124769

[B37] Iwai H. Tarao A. Satoh S. (2013). Changes in distribution of cell wall polysaccharides in floral and fruit abscission zones during fruit development in tomato (*Solanum lycopersicum*). *J. Plant Res.* 126 427–437. 10.1007/s10265-012-0536-0 23124772

[B38] Jensen T. E. Valdovinos J. G. (1967). Fine structure of abscission zones I. Abscission zones of the pedicels of tobacco and tomato flowers at anthesis. *Planta* 77 298–318. 10.1007/BF00389317 24522606

[B39] Jinn T.-L. Stone J. M. Walker J. C. (2000). HAESA, an *Arabidopsis* leucine-rich repeat receptor kinase, controls floral organ abscission. *Genes Dev.* 14 108–117. 10640280 PMC316334

[B40] Kalaitzis P. Solomos T. Tucker M. L. (1997). Three different polygalacturonases are expressed in tomato leaf and flower abscission, each with a different temporal expression pattern. *Plant Physiol.* 113 1303–1308. 10.1104/pp.113.4.1303 9112778 PMC158253

[B41] Kim J. (2014). Four shades of detachment: regulation of floral organ abscission. *Plant Signal. Behav.* 9 e976154. 10.4161/15592324.2014.976154 PMC4623469 25482787

[B42] Kim J. Dotson B. Rey C. Lindsey J. Bleecker A. B. Binder B. M. (2013). New clothes for the jasmonic acid receptor COI1: delayed abscission, meristem arrest and apical dominance. *PLoS ONE* 8:e60505 10.1371/journal.pone.0060505 PMC3613422 23573263

[B43] Kim J. Patterson S. E. (2006). Expression divergence and functional redundancy of polygalacturonases in floral organ abscission. *Plant Signal. Behav.* 1 281–283. 10.4161/psb.1.6.3541 19704626 PMC2634239

[B44] Kim J. Shiu S.-H. Thoma S. Li W.-H. Patterson S. E. (2006). Patterns of expansion and expression divergence in the plant polygalacturonase gene family. *Genome Biol.* 7 R87 10.1186/gb-2006-7-7-323 PMC1794546 17010199

[B45] Klee H. J. (2002). Control of ethylene-mediated processes in tomato at the level of receptors. *J. Exp. Bot.* 53 2057–2063. 10.1093/jxb/erf062 12324529

[B46] Konishi S. Izawa T. Lin S. Y. Ebana K. Fukuta Y. Sasaki T. (2006). An SNP caused loss of seed shattering during rice domestication. *Science* 312 1392–1396. 10.1126/science.1126410 16614172

[B47] Kwon-Ndung E. H. Misari S. M. (1999). “Overview of research and development of fonio (*Digitaria exilis* Kippis Stapf) and prospects for genetic improvement in Nigeria,” in *Genetics and Food Security in Nigeria* (Nigeria: GSN Publication), 71–76.

[B48] Lanahan M. B. Yen H. C. Giovannoni J. J. Klee H. J. (1994). The Never ripe mutation blocks ethylene perception in tomato. *Plant Cell* 6 521–530. 10.1105/tpc.6.4.521 8205003 PMC160455

[B49] Lashbrook C. C. Cai S. (2008). Cell wall remodeling in *Arabidopsis* stamen abscission zones: temporal aspects of control inferred from transcriptional profiling. *Plant Signal. Behav.* 3 733–736. 10.4161/psb.3.9.6489 19704843 PMC2634574

[B50] Leslie M. E. Lewis M. W. Youn J.-Y. Daniels M. J. Liljegren S. J. (2010). The EVERSHED receptor-like kinase modulates floral organ shedding in *Arabidopsis*. *Development* 137 467–476. 10.1242/dev.041335 20081191 PMC2858908

[B51] Lewis M. W. Leslie M. E. Liljegren S. J. (2006). Plant separation: 50 ways to leave your mother. *Curr. Opin. Plant Biol.* 9 59–65. 10.1016/j.pbi.2005.11.009 16337172

[B52] Li C. Zhou A. Sanng T. (2006). Rice domestication by reducing shattering. *Science* 311 1936–1939. 10.1126/science.1123604 16527928

[B53] Liljegren S. J. (2012). Organ abscission: exit strategies require signals and moving traffic. *Curr. Opin. Plant Biol.* 15 670–676. 10.1016/j.pbi.2012.09.012 23047135

[B54] Liljegren S. J. Leslie M. E. Darnielle L. Lewis M. W. Taylor S. M. Luo R. (2009). Regulation of membrane trafficking and organ separation by the NEVERSHED ARF-GAP protein. *Development* 136 1909–1918. 10.1242/dev.033605 19429787 PMC2680113

[B55] Ma C. Meir S. Xiao L. Tong J. Liu Q. Reid M. S. (2015). A KNOTTED1-LIKE HOMEOBOX protein regulates abscission in tomato by modulating the auxin pathway. *Plant Physiol.* 167 844–853. 10.1104/pp.114.253815 25560879 PMC4348773

[B56] Mao L. Begum D. Chuang H. W. Budiman M. A. Szymkowiak E. J. Irish E. E. (2000). JOINTLESS is a MADS-box gene controlling tomato flower abscission zone development. *Nature* 406 910–913. 10.1038/35022611 10972295

[B57] McKim S. M. Stenvik G. E. Butenko M. A. Kristiansen W. Cho S. K. Hepworth S. R. (2008). The BLADE-ON-PETIOLE genes are essential for abscission zone formation in *Arabidopsis*. *Development* 135 1537–1546. 10.1242/dev.012807 18339677

[B58] Meng X. Wang H. He Y. Liu Y. Walker J. C. Torii K. U. (2012). A MAPK cascade downstream of erecta receptor-like protein kinase regulates *Arabidopsis* inflorescence architecture by promoting localized cell proliferation. *Plant Cell* 24 4958–4960. 10.1105/tpc.112.104695 PMC3556968 23263767

[B59] Nakano T. Fujisawa M. Shima Y. Ito Y. (2013). Expression profiling of tomato pre-abscission pedicels provides insights into abscission zone properties including competence to respond to abscission signals. *BMC Plant Biol.* 13:40 10.1186/1471-2229-13-40 PMC3600680 23497084

[B60] Niederhuth C. E. Cho S. K. Seitz K. Walker J. C. (2013). Letting go is never easy: abscission and receptor-like protein kinases. *J. Integr. Plant Biol.* 55 1251–1263. 10.1111/jipb.12116 24138310

[B61] Ogawa M. Kay P. Wilson S. Swain S. M. (2009). *ARABIDOPSIS* DEHISCENCE ZONE POLYGALACTURONASE1 (ADPG1), ADPG2, and QUARTET2 are polygalacturonases required for cell separation during reproductive development in *Arabidopsis*. *Plant Cell* 21 216–233. 10.1105/tpc.108.063768 19168715 PMC2648098

[B62] Osborne D. J. (1989). Abscission. *Crit. Rev. Plant Sci.* 8 103–129. 10.1080/07352688909382272

[B63] Patterson S. E. (2001). Cutting loose. Abscission and dehiscence in *Arabidopsis*. *Plant Physiol.* 126 494–500. 10.1104/pp.126.2.494 11402180 PMC1540116

[B64] Patterson S. E. Bleecker A. B. (2004). Ethylene-dependent and –independent processes associated with floral organ abscission in *Arabidopsis*. *Plant Physiol.* 134 194–203. 10.1104/pp.103.028027 14701913 PMC316299

[B65] Plants and Society (2006). *Estelle Levetin and Karen McMahon*, 5th Edn New York City, NY: McGraw Hill, 184–236.

[B66] Polashock J. Zelzion E. Fajardo D. Zalapa J. Georgi L. Bhattacharya D. (2014). The American cranberry: first insights into the whole genome of a species adapted to bog habitat. *BMC Plant Biol.* 14:165 10.1186/1471-2229-14-165 PMC4076063 24927653

[B67] Pourkheirandish M. Hensel G. Kilian B. Senthil N. Chen G. Sameri M. (2015). Evolution of the grain dispersal system in barley. *Cell* 162 527–539. 10.1016/j.cell.2015.07.002 26232223

[B68] Rast M. I. Simon R. (2008). The meristem-to-organ boundary: more than an extremity of anything. *Curr. Opin. Genet. Dev.* 18 287–294. 10.1016/j.gde.2008.05.005 18590819

[B69] Roberts J. A. Elliott K. A. Gonzalez-Carranza Z. H. (2002). Abscission, dehiscence, and other cell separation processes. *Annu. Rev. Plant Biol.* 53 131–158. 10.1146/annurev.arplant.53.092701.180236 12221970

[B70] Roberts J. A. Whitelaw C. A. Gonzalez-Carranza Z. H. McManus M. T. (2000). Cell separation processes in plants: models, mechanisms, and manipulation. *Ann. Bot.* 86 223–235. 10.1006/anbo.2000.1203

[B71] Romero E. D. (2015). *Sunpreme: The Grape That Could Revolutionize The Raisin Industry, NPR.* Available at: http://www.npr.org/sections/thesalt/2015/10/07/446590533/sunpreme-the-grape-that-could-revolutionize-the-raisin-industry

[B72] Sang T. (2009). Genes and mutations underlying domestication transitions in grasses. *Plant Physiol.* 149 63–70. 10.1104/pp.108.128827 19126696 PMC2613738

[B73] Sawicki M. Barka E. A. Clément C. Vaillant-Gaveau N. Jacquard C. (2015). Cross-talk between environmental stresses and plant metabolism during reproductive organ abscission. *J. Exp. Bot.* 66 1707–1719. 10.1093/jxb/eru533 25711702 PMC4669552

[B74] Sexton R. Roberts J. A. (1982). Cell biology of abscission. *Annu. Rev. Plant Physiol.* 33 133–162. 10.1146/annurev.pp.33.060182.001025

[B75] Shi C.-L. Stenvik G.-E. Vie A. K. Bones A. M. Pautot V. Proveniers M. (2011). *Arabidopsis* class I KNOTTED-like homeobox proteins act downstream in the IDA-HAE/HSL2 floral abscission signaling pathway. *Plant Cell* 23 2553–2567. 10.1105/tpc.111.084608 21742991 PMC3226213

[B76] Singh A. P. Tripathi S. K. Nath P. Sane A. P. (2011). Petal abscission in rose is associated with the differential expression of two ethylene-responsive xyloglucan endotransglucosylase/hydrolase genes, RbXTH1, and RbXTH2. *J. Exp. Bot.* 62 5091–5103. 10.1093/jxb/err209 21765161 PMC3193013

[B77] Stenvik G. Tandstad N. Guo Y. Shi C. (2008). The EPI Peptide of INFLORESCENCE DEFICIENT IN ABSCISSION is sufficient to induce abscission in *Arabidopsis* through the receptor-like kinases HAESA and HAESA-LIKE2. *Plant Cell* 20 1805–1817. 10.1105/tpc.108.059139 18660431 PMC2518227

[B78] Sundaresan S. Philosoph-Hadas S. Riov J. Belausov E. Kochanek B. Tucker M. L. (2015). Abscission of flowers and floral organs is closely associated with alkalization of the cytosol in abscission zone cells. *J. Exp. Bot.* 66 1355–1368. 10.1093/jxb/eru483 25504336 PMC4339595

[B79] Tekete M. L. (2006). *Etude du Photopériodisme du Fonio*. Ph.D. thesis, Diplôme d’Ingénieur Agronome de l’Institut Polytechnique Rural de Formation et de Recherche Appliquée de Katibougou (IER), French.

[B80] Van Nocker S. (2009). Development of the abscission zone. *Stewart Postharvest Rev.* 5 1–6. 10.2212/spr.2009.1.5

[B81] Wang X. Xu W. H. Ma L. Fu Z. Deng X. Li J. (2006). Requirement of KNAT1/BP for the development of abscission zones in *Arabidopsis thaliana*. *J. Integr. Plant Biol.* 48 15–26. 10.1111/j.1744-7909.2005.00085.x-i1

[B82] Wei P.-C. Tan F. Gao X.-Q. Zhang Z.-Q. Wang G.-Q. Xu H. (2010). Overexpression of AtDOF4.7, an *Arabidopsis* DOF family transcription factor, induces floral organ abscission deficiency in *Arabidopsis*. *Plant Physiol.* 153 1031–1045. 10.1104/pp.110.153247 20466844 PMC2899910

[B83] Zhou Y. Lu D. Li C. Luo J. Zhu B. F. Zhu J. (2012). Genetic control of seed shattering in rice by the APETALA2 transcription factor shattering abortion1. *Plant Cell* 24 1034–1048. 10.1105/tpc.111.094383 22408071 PMC3336138

[B84] Zohary D. Hopf M. (2000). *Domestication of Plants in the Old World*, 3rd Edn Oxford: Oxford University Press, 16–91.

