# Supplementary material for: Are We on the Right Track: Can Our Understanding of Abscission in Model Systems Promote or Derail Making Improvements in Less Studied Crops?
Source: Front Plant Sci. 2016 Jan 26;6:1268. doi: 10.3389/fpls.2015.01268 (PMC4726918; doi:10.3389/fpls.2015.01268)
Supplement: Supplementary file 1 [file Table_1.DOCX]

Supplemental Table 1

| **Name of Gene and Initial Characterization** | **Grape**  *Vitis vinifera*  (%identity/%similarity) | **Cranberry**  *Vaccinium macrocarpon*  (% identity) | **Tomato**  *Lycopersicum lycopersicum*  (%identity/%similarity) |
| --- | --- | --- | --- |
| ***AGAMOUS-LIKE -15***  ***(AGL-15) (MADS box)***  **At5g13790**  Fernandez et al., 2000 | unnamed protein product  GI:296085099 (55/74)  and  PREDICTED: agamous-like MADS-box protein AGL15  GI:731414618 (55/73) | Contig40100 identity 72%  Fajardo et al (2014)  g19544.t1 identity 81%  Contig 274030 identity 74%  Polashock et al. (2014) | MADS-box transcription factor 3  Solyc01g087990 (50/64) |
| **BREVIPEDICELLUS /KNOTTED-LIKE FROM ARABIDOPSIS THALIANA 1**  **BP/KNAT1 (homeobox)**  **At4g08150**  Wang et al., 2006 | PREDICTED: homeobox protein knotted-1-like 2  GI:225458942 (61/74) | contig02342 Identity 82% contig131171 Identity 80.00% Fajardo et al (2014)  g8128.t1 identity 80%  contig_436569 identity 82%  Polashock et al. (2014) | Solyc04g077210  (62.3/83.1) |
| ***CELLULASE 5***  ***CEL5* (cell wall degrading enzyme)**  **At1G22880**  delCampillo, 1999 | PREDICTED: endoglucanase 3  GI:359493565 (76/85) | g5250.t1 identity 66% g7812.t1 Identity 67% contig_2165129 identity 66%  Polashock et al. (2014)  contig11883 Identity 71%  contig17117 Identity 71.40% Fajardo et al. (2014) | Solyc08g083210  (58/84) |
| ***CORONATINE INSENSITIVE 1 (Jasmonic acid receptor)***  ***COI1***  **At2g39940**  Kim *et al*., 2013 | Coronatine insensitive  protein 1  GI:359486723 (72/83)  and  GI:381141436 (72/83) | g1943.t1  Identity 71%  Polashock et al. (2014)  Contig03141 Identity 67%  Fajardo et al. (2014) | Solyc05g052620  (69/87) |
| ***SECRETORY CARRIER-ASSOCIATED MEMBRANE PROTEIN 5/DELAYED ABSCISSION 5***  ***DAB5 /SCAMP5***  **At1g32050**  Smith, 2010 | secretory carrier-associated membrane proteins  too many matches* | contig139734 Identity 81%  Fajardo et al. (2014) | Solyc08g076210  (71/82) |
| ***ETHYLENE INSENSITIVE 2***  ***EIN2***  **At5g03280**  Patterson and Bleecker, 2004 | **PREDICTED: ethylene-insensitive protein 2**  GI:225440009 (57/71) | g16249.t1 Identity 69%  g5834.t1 Identity 67% contig_314863 identity 66% contig_174939 Identity 69% Polashock et al. (2014) | Solyc09g007870  (50/75) |
| ***ETHYLENE INSENSITIVE 3***  ***EIN3***  **At3g20770**  Chao et al., 1997 | too many matches* | too many matches*  (both databases) | NO match NCBI |
| *ETHYLENE RESPONSE1*  *ETR1* ***(receptor-like kinase)***  At1g66340  Chang et al., 1993 | RECEPTOR-LIKE KINASES  too many matches* | \| too many matches* \| \| --- \| \| (both databases) \| | RECEPTOR-LIKE KINASES  too many matches* |
| *EVERSHED*  *EVR* ***(receptor-like kinase)***  **SUPPRESSOR OF BIR1-1**  [AT2G31880](http://www.arabidopsis.org/servlets/TairObject?type=locus&name=AT2G31880)  Leslie at al., 2010 | PREDICTED: leucine-rich repeat receptor-like serine/threonine/tyrosine-protein kinase SOBIR1  GI:225456834 (65/78) | \| too many matches* \| \| --- \| \| (both databases) \| | RECEPTOR-LIKE KINASES  too many matches* |
| ***HAESA/RECEPTOR-LIKE KINASE5***  ***HAE/RLK5***  **At4g28490**  Jinn *et al*., 2000 | RECEPTOR-LIKE KINASES  too many matches* | \| too many matches* \| \| --- \| \| (both databases) \| | RECEPTOR-LIKE KINASES  too many matches* |
| ***HAESA-LIKE2***  ***HSL2 (receptor-like kinase)***  **At5g65710**  Shi et al., 2011 | RECEPTOR-LIKE KINASES  too many matches* | contig53545 Identity 67% Fajardo et al (2014)  g30811.t1 Identity 66% contig_331423 Identity 67% Polashock et al. (2014) | RECEPTOR-LIKE KINASES  too many matches* |
| ***HAWAIIAN SKIRT***  ***HWS (F-box)***  **At3g61590**  Gonzalez-Carranza *et al*., 2007 | PREDICTED: F-box/kelch-repeat protein  GI:225454416 (65/80) | contig58110 Identity 81%  Fajardo et al (2014)  g4586.t1 identity 69%  contig_2151670 identity 72%  Polashock et al. (2014) | Solyc01g095370  (65.3/85.4) |
| ***INFLORESCENCE DEFICIENT IN ABSCISSION***  ***IDA (putative receptor ligand)***  **At1g68765**  Butenko *et al*., 2003 | IDA-LIKE 4-like LOC GI:731388038  41/65 | contig74046 identity 92%  contig27538 identity 92%  Fajardo et al (2014) | Solyc05g010000  (48/81) |
| ***KNOTTED-LIKE FROM ARABIDOPSIS THALIANA 6***  ***KNAT6***  ***(Homeobox)***  **At1g23380**  Chun-Lin et al., 2011 | PREDICTED: homeobox protein knotted-1-like 6  GI:225425603 (69/82) | HR contig02342 identity 78%  Fajardo et al (2014)  g8128.t1 identity 76%  g27567.t1 identity 74%  contig_454157 identity 74%  Polashock et al. (2014) | Solyc05g005090  54.6/75.5 |
| ***NEVERSHED***  ***NEV***  ***(ADP-ribosylation GTPase)***  ***At5g54310***  Liljegren et al., 2009 | PREDICTED: probable ADP-ribosylation factor GTPase-activating protein AGD5  GI:359493891 (59/69) | contig29957 identity 85%  Fajardo et al (2014)  g17865.t1 identity 67%  g6436.t1 identity 82%  contig_145748 identity  83%  Polashock et al. (2014) | probable ADP-ribosylation factors  too many matches* |

**Supplemental Table 1. Examples of Genes Identified in Arabidopsis and orthologs from grape, cranberry and tomato.** The table illustrates the complexity of identifying the appropriate orthologous genes based on e value, and percent identity and similarity, as many genes have more than five choices (indicated by *). Note that several of the kinase genes including *ETR1* and *HAESA,* the *SCAMPS,* and *EIN3* had so many matches that we were unable to select the best ortholog. Grape orthologs were identified using NCBI Basic Local Alignment Search Tool blastp. Cranberry genes/contigs were identified using the two databases and hits are referenced accordingly. Tomato orthologs were identified using the Sol database from the Sol Genomics network <http://solgenomics.net/>. Protein sequences were also queried using NCBI for grape; however, the limited sequence availability did not provide any useful information.
